# Random mutagenesis analysis and identification of a novel C_2_H_2_-type transcription factor from the nematode-trapping fungus *Arthrobotrys oligospora*

**DOI:** 10.1038/s41598-017-06075-5

**Published:** 2017-07-17

**Authors:** Dewei Jiang, Jing Zhou, Guizhen Bai, Xinjing Xing, Liyan Tang, Xuewei Yang, Juan Li, Ke-Qin Zhang, Jinkui Yang

**Affiliations:** 1 State Key Laboratory for Conservation and Utilization of Bio-Resources in Yunnan, Yunnan University, Kunming, 650091 P. R. China; 2 Key Laboratory of Animal Models and Human Disease Mechanisms of the Chinese Academy of Sciences & Yunnan Province, Kunming Institute of Zoology, Chinese Academy of Sciences, Kunming, 650223 P. R. China; 3 Panzhihua Institute for Food and Drug Control, Panzhihua, 617000 P. R. China

## Abstract

*Arthrobotrys oligospora* is a typical nematode-trapping fungus. In this study, 37 transformants of *A. oligospora* were obtained by REMI (restriction enzyme mediated integration) method and phenotypic properties of nine transformants were analyzed. The nine transformants showed differences in growth, conidiation, trap formation, stress tolerance, and/or pathogenicity among each other and with those of the parental wild-type strain (WT). The insertional sites of the *hph* cassette were identified in transformants X5 and X13. In X5, the cassette was inserted in the non-coding region between *AOL_s00076g273* (*76g273*) and *AOL_s00076g274* (*76g274*) and the transcription of *76g274*, but not *76g273*, was enhanced in X5. 76g274p had two conserved domains and was predicted as a nucleoprotein, which we confirmed by its nuclear localization in *Saccharomyces cerevisiae* using the green fluorescent protein-fused 76g274p. The transcription of *76g274* was stimulated or inhibited by several environmental factors. The sporulation yields of *76g274*-deficient mutants were decreased by 70%, and transcription of several sporulation-related genes was severely diminished compared to the WT during the conidiation. In summary, a method for screening mutants was established in *A. oligospora* and using the method, we identified a novel C_2_H_2_-type transcription factor that positively regulates the conidiation of *A. oligospora*.

## Introduction

Nematode-trapping fungi (NTF) are an unique group of organisms broadly distributed in terrestrial and aquatic ecosystems throughout the world^1, 2^. Most NTF can live both saprophytically on dead organic matter and as predators by capturing tiny animals^1, 2^. These fungi can capture nematodes by producing specific mycelia structures (traps) and then obtain nutrients from their nematode prey^3–6^. Their broad adaptability and flexible lifestyles make them potential agents for controlling parasitic nematodes of plants and animals^2^. However, it is difficult to perform genetic manipulation in NTF due to their multi-nuclei nature in a single cell. The multi-nuclei nature of NTF has been observed using DAPI and Hoechst 33258 staining, the number of nuclei in a conidium of NTF can vary significantly^7, 8^. For example, 9–16 nuclei were observed in the conidium of *Arthrobotrys oligospora* stained by Hoechst 33258, similarly, 4–17 and 10–19 nuclei were observed in *A. amerospora* and *A. conoides*, respectively^7^. While 12 nuclei were observed in *A. amerospora* stained by DAPI, and 6–20 nuclei were observed in *A. conoides* ^8^. In the past 50 years, researchers have made great efforts in identifying pathogenicity-related genes^4–6, 9^, and in understanding the difference between vegetative mycelia and traps^10–13^. Recently, three species of NTF producing different traps were individually sequenced^11, 12, 14^, in which the trap cell proteome of the fungus *M. haptotylum* ^15^, and the proteomic and transcriptional analyses of cell wall related proteins of *Arthrobotrys oligospora* ^16^, were characterized. The information from the NTF genomes, proteomes and transcriptomes has contributed to identifying the important genes involved in the regulation of mycelial development, conidiation, trap formation and pathogenicity.

Construction of random mutant library is a useful method for identifying genes of unknown functions in microorganisms. Several methods have been developed and reported in different fungal species, such as restriction enzyme mediated integration (REMI), transposon arrayed gene knockout (TAGKO) and *Agrobacterium tumefaciens*-mediated transformation (ATMT)^17^. TAGKO has been reported in diverse fungi, such as *Fusarium oxysporum* ^18^ and *Aspergillus niger* ^19^. ATMT has been widely applied in genetic transformation of filamentous fungi, such as *Magnaporthe grisea* ^20^, *Ustilago maydis* ^21^ and *Cordyceps militaris* ^22^. REMI technique was initially established in *Saccharomyces cerevisiae* ^23^ and has been widely used in diverse fungi. In 1999, the hygromycin-B phosphotransferase gene (*hph*) was transformed to *A. oligospora* using REMI method, and 7 out of 13 transformants were single copy integrations, whereas the remaining were multiple and more complex integrations^24^. Subsequently, the encoding gene of serine protease PII^6^ was transformed into *A. oligospora* by REMI method, and mutants containing additional copies of the *PII* gene developed a higher number of infection structures and had an increased speed of capturing and killing nematodes than the wild type strain^9^. Recently, an improved REMI method was applied to transform *A. oligospora* ^25^ and *Monacrosporium sphaeroides* ^26^. REMI method can reliably obtain considerable mutant frequencies and single-copy insertion rates compared to TAGKO and ATMT, although the transformation utilizing protoplasts limits its application in fungi^17^.

*A. oligospora* can capture nematodes by producing three-dimensional networks^2^. The fungus has been a model species for studying the interaction between NTF and nematodes. Recently, the genome of *A. oligospora* was sequenced; it contains 40.07 Mb assembled sequence with 11,479 predicted genes, and only 44.6% were mapped in the KOG/COG database^14^. Comparative genomic analysis showed that 53.74% genes are specific to *A. oligospora* ^14^. While the majority of these genes had not been annotated, we hypothesize that at least some of them might be involved in the unique biological functions in this fungus, such as trap formation and conidiation. In order to identify novel genes related to trap formation, conidiation and virulence in *A. oligospora*, 37 random-insertional mutants were screened by REMI method, and nine of them were selected for comparing their physiological and biochemical properties. Furthermore, the insertional sites of *hph* cassette in the mutants X5 and X13 were identified, and a novel Zinc-finger protein was characterized in *A. oligospora*.

## Results

### Random mutagenesis of *A. oligospora* by REMI technique

The size of the recombinational vector (pMD-hph) is 4.1 kb, including 7 common single restriction sites located in the 5′ and 3′ ends of the *hph* cassette (Supplementary Fig. S1), recognized by enzymes XbaI, KpnI, SmaI, XmaI, SacI, SalI and HindIII. The protoplasts of *A. oligospora* were prepared according to methods described previously^24, 27^, and 1 × 10^7^−10^8^ protoplasts/mL was used for transformation. The sensitivity of *A. oligospora* against hygromycin B (Hyg, 200 µg/mL) was used to screen the transformants.

The rate of endonuclease-induced damage to protoplasts was determined using restriction enzymes XbaI (X group) and SmaI (A group), respectively, and 30 U was determined as a suitable concentration for REMI transformation (Supplementary Table S1). In total, 41 (X group) and 7 (A group) transformants were screened and identified (Supplementary Table S1), respectively. Those Hyg-resistant (Hyg^r^) transformants were verified by amplifying the *hph* cassette and five of them were false-positive transformants. Then six Hyg^r^ strains lost the *hph* cassette after continuous sub-culture on screening medium, thus the genetic stability of the transformants was determined to be 86%, consistent with that in previous reports^25, 26^.

### Physiological and biochemical analysis of mutants

**Figure 1:**
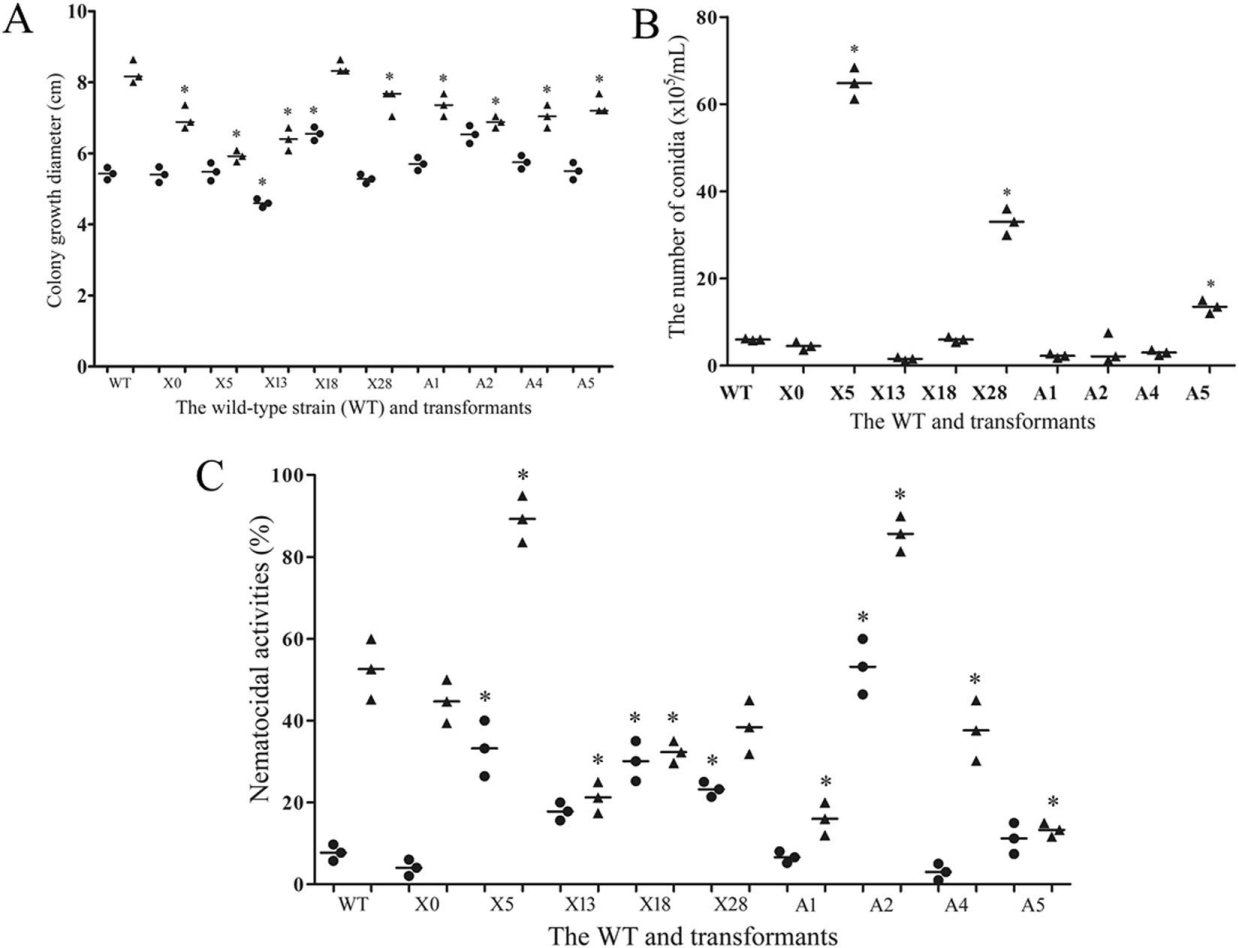
Phenotypic properties of the WT and nine transformants. (**A**) The growth rates of WT and transformants on TYGA (dots) and PDA (triangles) plates. *P < 0.05 versus WT. (**B**) Conidiation of WT and transformants on CMA plate at 26 °C for 2 weeks. *P < 0.05 versus WT. C. Comparison on the nematocidal activities of WT and transformants on WA (water agar) plate. *P < 0.05 versus WT at 10 h (dots) and 24 h (triangles), respectively. The horizontal bars depict the median, and data are presented as suggested by Weissgerber *et al*.^58^ and Klaus^59^.

Nine mutants were randomly chosen to determine their phenotypic and physiological properties. Differences in mycelial growth were observed among the mutants and the wild-type strain (WT) in TYGA and PDA (Fig. 1A). In general, the fungi grew faster in PDA than that in TYGA. Mutants X18 and A2 grew faster than the WT in the TYGA, while X13 grew slower than the WT, and other mutants had no difference to the WT. The growth of X18 was similar to the WT in PDA, while other mutants grew significantly slower than the WT (Fig. 1A). Meanwhile, the conidiation of mutants also showed obvious change compared to the WT, with the conidial production of the strains X5, X28 and A5 increased by 10.8, 5.5 and 2.3 folds (Fig. 1B), respectively. On the contrary, the conidial production of the strains X13, A1 and A2 were decreased by 74%, 62% and 66%, respectively, compared to the WT. Moreover, the conidial germination rate of all mutants was observed to have no significant difference from that of WT. Each conidium of strains X5 and X13 produced one germ tube, same as the WT. However, several mutants (such as A1, A2, X28, A5 and X0) produced two germ tubes from a single conidium, while some mutants had conidia producing either one or two germ tubes, such as A4 and X18 (Supplementary Fig. S2). After cultured in LMZ for 6 days, the fermentation liquid of the WT showed obvious protease activity, while the proteolytic activity of mutants such as A1, A4 and X0 was decreased, and the other six mutants had no protease activity (Supplementary Fig. S3).

The WT and eight of the nine mutants could produce traps after induced by nematodes on water agar (WA) plates, while mutant X13 did not produce any trap (Supplementary Fig. S4). The nematicidal activity of the WT was 9% and 52% when induced by nematodes for 10 and 24 h (Fig. 1C), respectively. However, the nematicidal activities of X5 and A2 were considerably increased, and 33% and 89% nematodes were killed by X5 when induced by nematodes for 10 and 24 h, respectively. On the contrary, several mutants, such as X13, A1 and A5, showed lower nematocidal activity than the WT when induced by nematodes for 24 h.

All mutants grew well on the TG plates, whereas their growths were inhibited when supplemented with NaCl, H_2_O_2_ and SDS in medium, similar to that of the WT strain. Four mutants (A2, A5, X0 and X13) could not grow in the TG medium supplemented with 0.05% SDS, while other five mutants (A1, A4, X5, X18 and X28) could grow slightly (Supplementary Fig. S5). Similarly, strains X5, X18 and X28 could grow on the TG medium supplemented with H_2_O_2_ (30 mM), but WT and other mutants could not grow on this medium. Moreover, all mutants could grow on the TG media supplemented with NaCl (0.3 M), but their growth rates were slower than those on the medium without NaCl. A2 grew slower than the WT, and the X18 grew faster than the WT in the TG medium supplemented with 0.3 M NaCl (Supplementary Fig. S5).

### Identification of the insertion sites of the *hph* cassette in mutants X5 and X13

**Figure 2:**
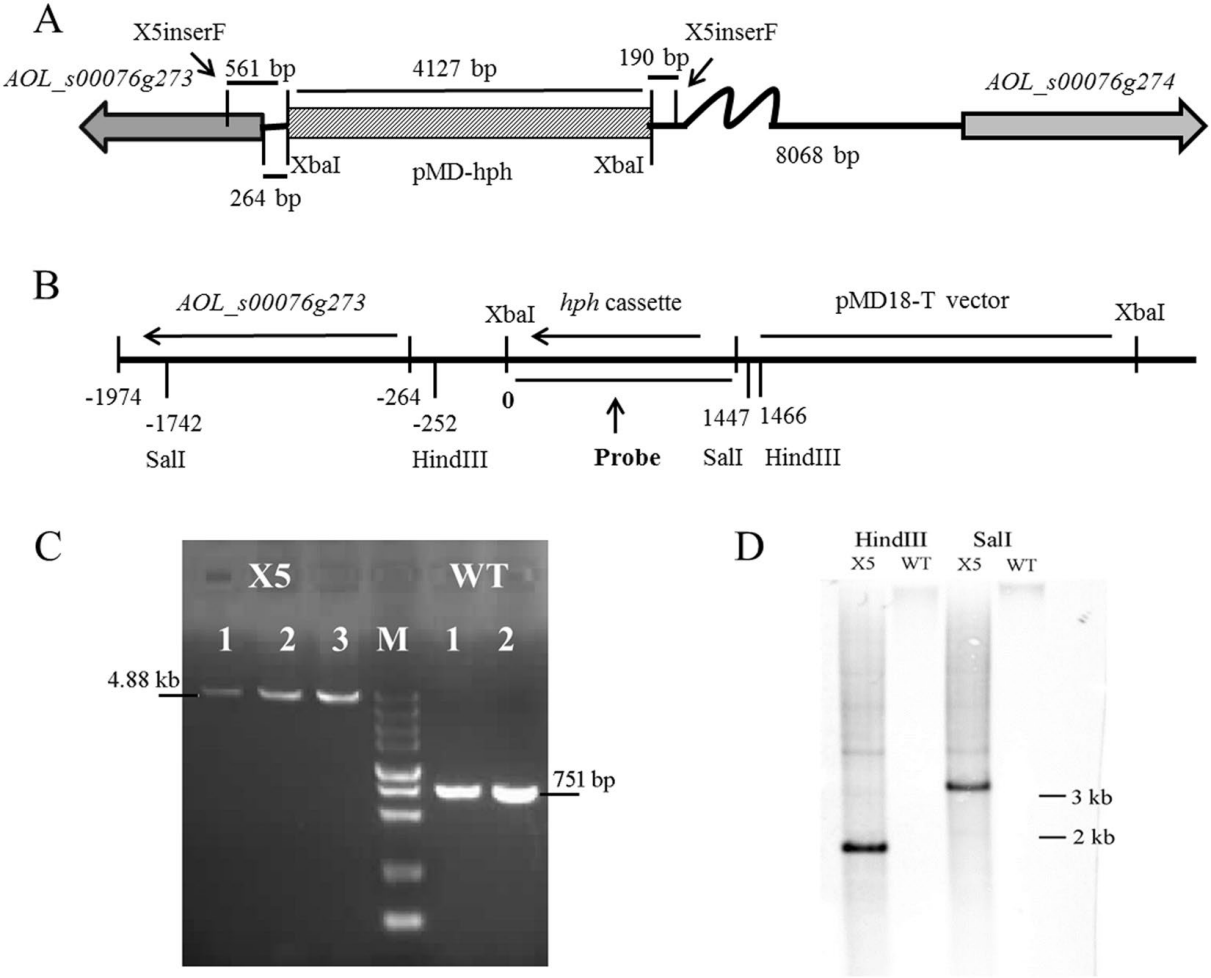
Identification and verification of the insertion of the *hph* cassette in transformants X5. (**A**) The sketch map of the insertion of the pMD-hph in transformants X5. The frame filled with diagonal lines presented the insertion of pMD-hph, and the X5inserF and X5inserR indicated the location of primers used for the PCR verification in Fig. 2C. (**B**) The schematic diagram of the location of gene *76g273* and the pMD-hph in X5, and restriction enzymes (SalI and HindIII) used for Southern blotting analysis were also indicated. The inserted site of the pMD-hph was set to 0, and the expected size of band hybridizing to the *hph* cassette in the X5 was 3189 bp and 1718 bp after digested by SalI and HindIII, respectively. (**C**) Verification of the insertion of the *hph* cassette in transformants X5 by PCR amplification using primers X5inserF/X5inserR. M, DNA marker, ladders are 5 kb, 3 kb, 2 kb, 1.5 kb, 1 kb, 750 bp, 500 bp, 250 bp and 100 bp. (**D**) Southern blotting analysis of the WT and X5 using restriction enzymes SalI and HindIII.

Flanking sequences of the *hph* cassette in strains X15 and X13 were amplified by DNA walking method, and the insertion sites were identified by blast analysis in the genome of *A. oligospora*. The *hph* cassette was determined to be inserted into the non-coding region (NCR) between *AOL_s00078g287* (*78g287*) and *AOL_s00078g288* (*78g288*) in X13 (Supplementary Fig. S6), and in the NCR between *AOL_s00076g273* (*76g273*) and *AOL_s00076g274* (*76g274*) in X5 (Fig. 2A). The insertion sites of the *hph* cassette in mutants X5 and X13 were further confirmed by PCR amplification using primers X5inserF/X5inserR and X13inserF/X13inserR (Supplementary Table S2), respectively. Specific bands of expected sizes were amplified from the WT and X5 (Fig. 2C), except that a weak 660 bp-band the same size as in the WT was also amplified from X13 (Supplementary Fig. S6). The 660 bp-band in X13 was sequenced and it was consistent with the sequence of the WT, which suggested that strain X13 lies in a heterozygotic state. Moreover, the insertion of *hph* cassette in the X5 was further analyzed by Southern blotting using two restriction enzymes, HindIII and SalI (Fig. 2B), respectively, which had only one recognition site in the recombinational vector pMD-hph (Fig. S1). The genomic DNA of the WT and X5 was digested with either HindIII or SalI, respectively, and the *hph* cassette was used as a probe for Southern analysis. A single and expected size of band hybridizing to the *hph* cassette was observed in the X5 after its genomic DNA was digested using HindIII or SalI, respectively, while no band was observed in the WT under same condition (Fig. 2D), which suggested that the *hph* cassette was a single integration in X5.

**Figure 3:**
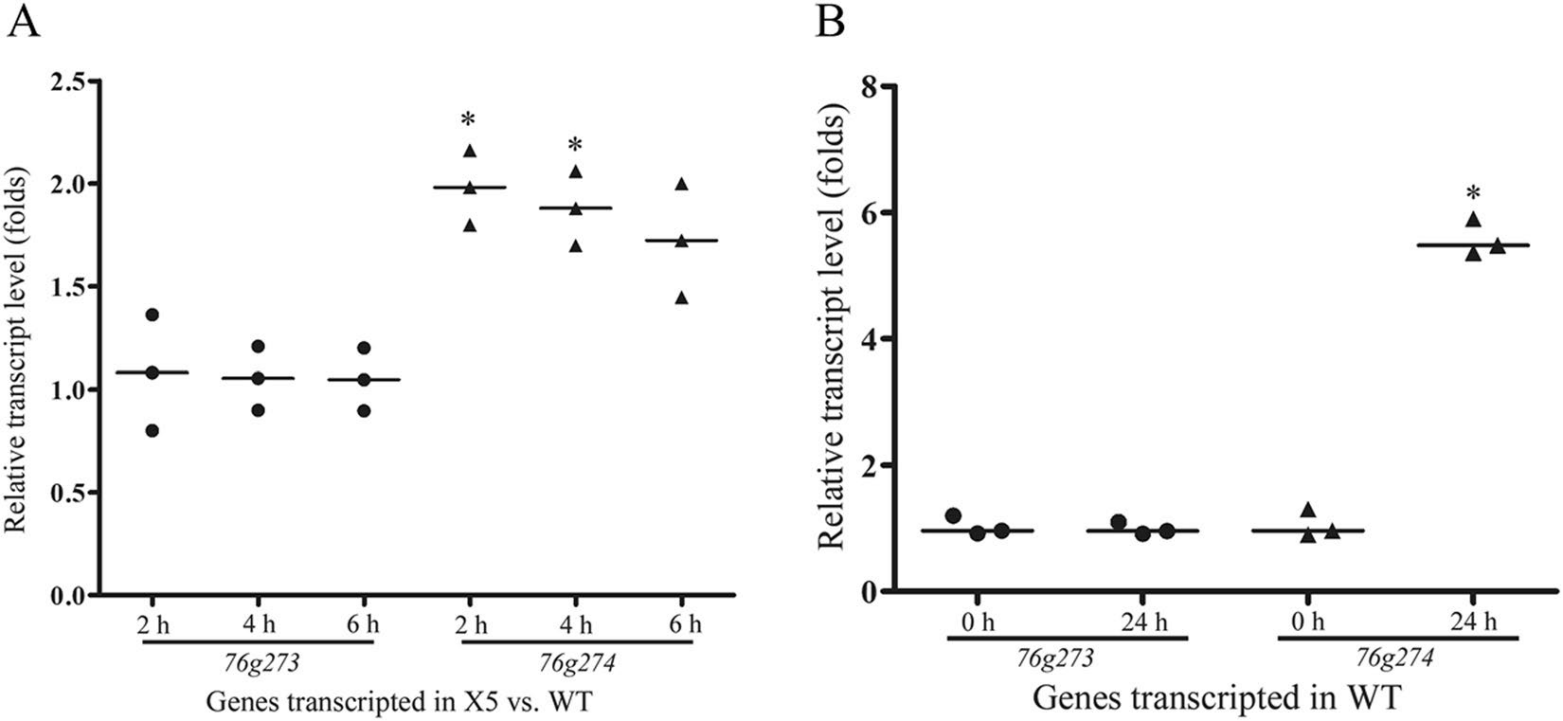
Transcriptional analysis of genes *76g273* and *76g274* in WT and X5. β-tubulin gene (AOL_s00076g640) of *A. oligospora* was used as the reference standard. (**A**) The transcription of *76g273* and *76g274* in WT and X5 at 2 d, 4 d and 6 d, respectively. *P < 0.05 versus WT. (**B**) The transcriptional levels of genes *76g273* and *76g274* in WT after induced by nematodes for 0 h and 24 h. *P < 0.05 versus 0 h. The horizontal bars depict the median, and data are presented as suggested by Weissgerber *et al*.^58^ and Klaus^59^.

The transcriptional levels of genes *76g273* and *76g274* were determined by real time PCR (RT-PCR) in WT and X5, respectively. There was about a two-fold increase of gene *76g274* in strain X5 compared to that of WT at 2, 4 and 6 days, respectively, while that of gene *76g273* showed no significant difference compared to that of WT (Fig. 3A). Moreover, the transcriptional levels of genes *76g273* and *76g274* were determined in the WT after induced by nematodes at 0 h and 24 h, respectively. Our results showed that the transcription of gene *76g274* was significantly up-regulated (*p* < 0.05) after induced by nematodes (Fig. 3B), which suggested that gene *76g274* might be involved in trap formation and infection process in *A. oligospora*.

### Analysis and prediction of the protein 76g274p

**Figure 4:**
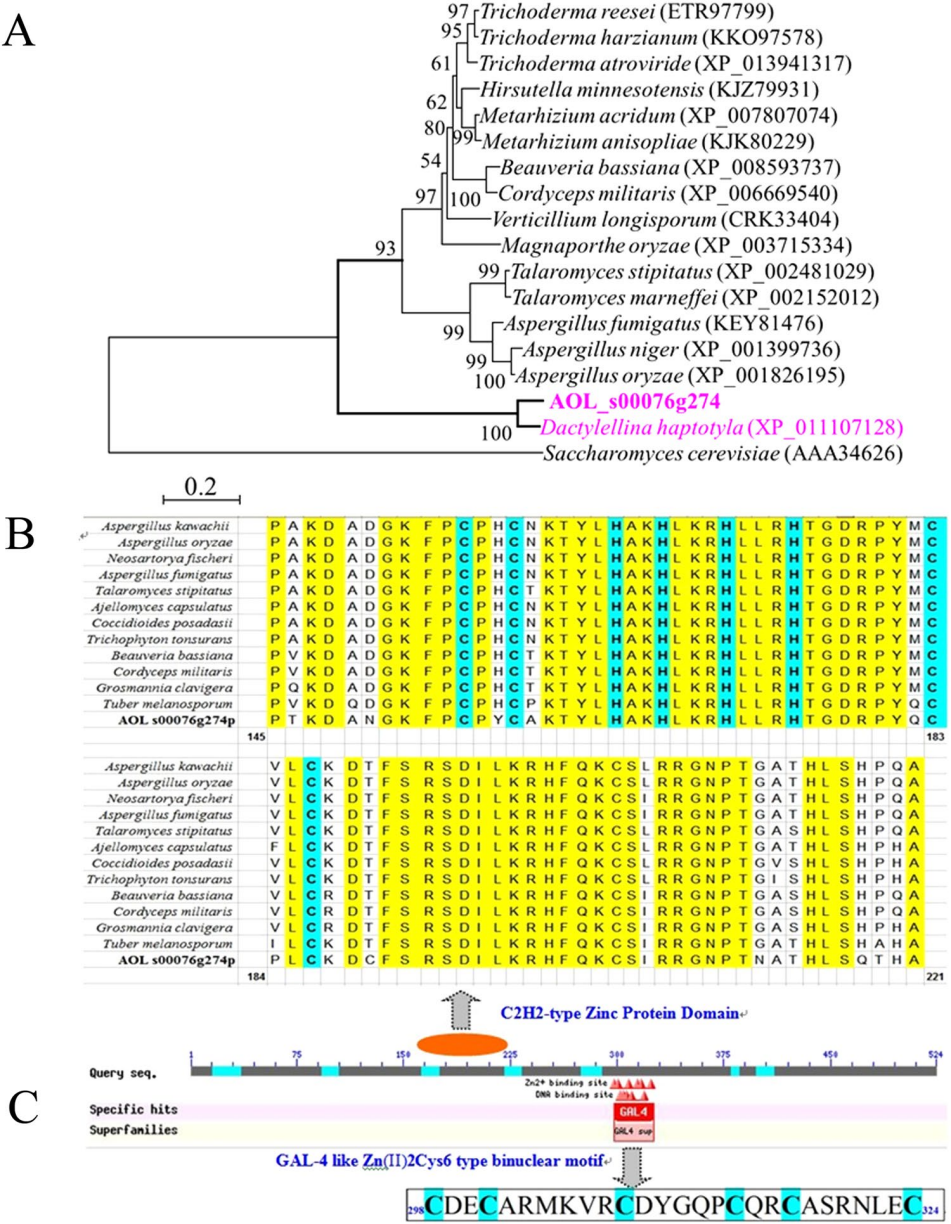
Phylogenetic analysis of 76g274p in different fungi and prediction of conserved domains of 76g274p. (**A**) Phylogenetic tree based on the amino acid sequence of C_2_H_2_-like proteins from different fungi. The number in the parentheses behind the fungal name is the GenBank number of the homologous proteins of 76g274p in different fungi. The 76g274p and its homologous protein from *D. haptotyla* were marked in red. Protein (AAA34626) from *Saccharomyces cerevisiae* used as an outgroup. (**B**) Comparison on the conserved Zinc finger C_2_H_2_-type domain of 76g274p and homologous proteins from different fungi. The conserved amino acid residues were marked in yellow, and C_2_H_2_ was marked in blue. (**C**) The conserved Zn(2)-C6 fungal-type DNA-binding domain in 76g274p. Six cysteine residues were marked in blue.

According to above analysis, gene *76g274* was chosen for further study. The gene *76g274* comprises of an open reading frame containing two introns and three exons. It codes for a polypeptide (76g274p) of 524 amino acid residues without a signal peptide, with a predicted isoelectric point and molecular weight of 9.43 and 57.5 kDa, respectively. Homologous analysis indicated that 76g274p shares a low sequence identity (26.6–35.0%) to analogues from other filamentous fungi, such as *Aspergillus fumigatus*, *Aspergillus niger*, *Beauveria bassiana*, *Magnaporthe oryzae* and *Trichoderma reesei*. Interestingly, 76g274p shares a high degree of sequence identity (86.5%) to a homologous protein from *Dactylellina haptotyla* (syn. *M. haptotylum*), a nematode-trapping fungus. Phylogenetic analysis also showed that the analogues of 76g274p from different filamentous fungi clustered into two clades (Fig. 4A). Protein 76g274p and its homologous protein from *D. haptotyla* were clustered in a single clade, suggesting that they share a close evolutionary relationship with each other but distant from other fungi.

The functional domains of 76g274p were analyzed using Interpro^28^, and two conserved domains (Zinc finger C_2_H_2_-type/integrase DNA-binding domain [IPR013087] and Zn(2)-C6 fungal-type DNA-binding domain [IPR001138]) were determined. Alignment analysis of 76g274p and other fungal analogues showed that they share a typical C_2_H_2_-type protein domain (Fig. 4B), however, they share a low identity (26.6–35.0%) on the whole sequences. The amino acid residues in position 145–221 of 76g274p was highly conservative among different fungi, among which the amino acid sequences at positions 155–167 and 171–186 were found to be two typical C_2_H_2_-type Zn^2+^-binding domains. Moreover, sequences in positions 298–324 consisted of the conserved Zn(2)-C6 fungal-type DNA-binding domain (Fig. 4C).

Other conserved features were predicted using PredictProtein^29^ and LOCtree^30^. The protein 76g274p was predicted to be located in the nucleus, but no nuclear localization sequence (NLS) similar to known NLS was identified. Additionally, a non-ordinary secondary structure region was predicted in the C-terminal of 76g274p, which was located at positions 395–524. Furthermore, several protein-protein interaction sites were predicted in 76g274p, such as the cAMP-/cGMP-dependent protein kinase phosphorylation site, a protein kinase C phosphorylation site, an N-myristoylation site, a casein kinase II phosphorylation site and a tyrosine kinase phosphorylation site. Analyses via Swiss-Model and STRING showed that a zinc-finger protein structure was located in the conserved domain of 76g274p at positions 151–205 and that it was similar to the zinc-finger domain 2 (no. cltf6D) from *Xenopus* TFIIIA^31^, which can bind to the 5 s ribosomal RNA gene internal control region. However, little information about the interaction partners of 76g274p was predicted by STRING, suggesting it might be a novel protein with unknown function.

### Localization analysis of the protein 76g274p

**Figure 5:**
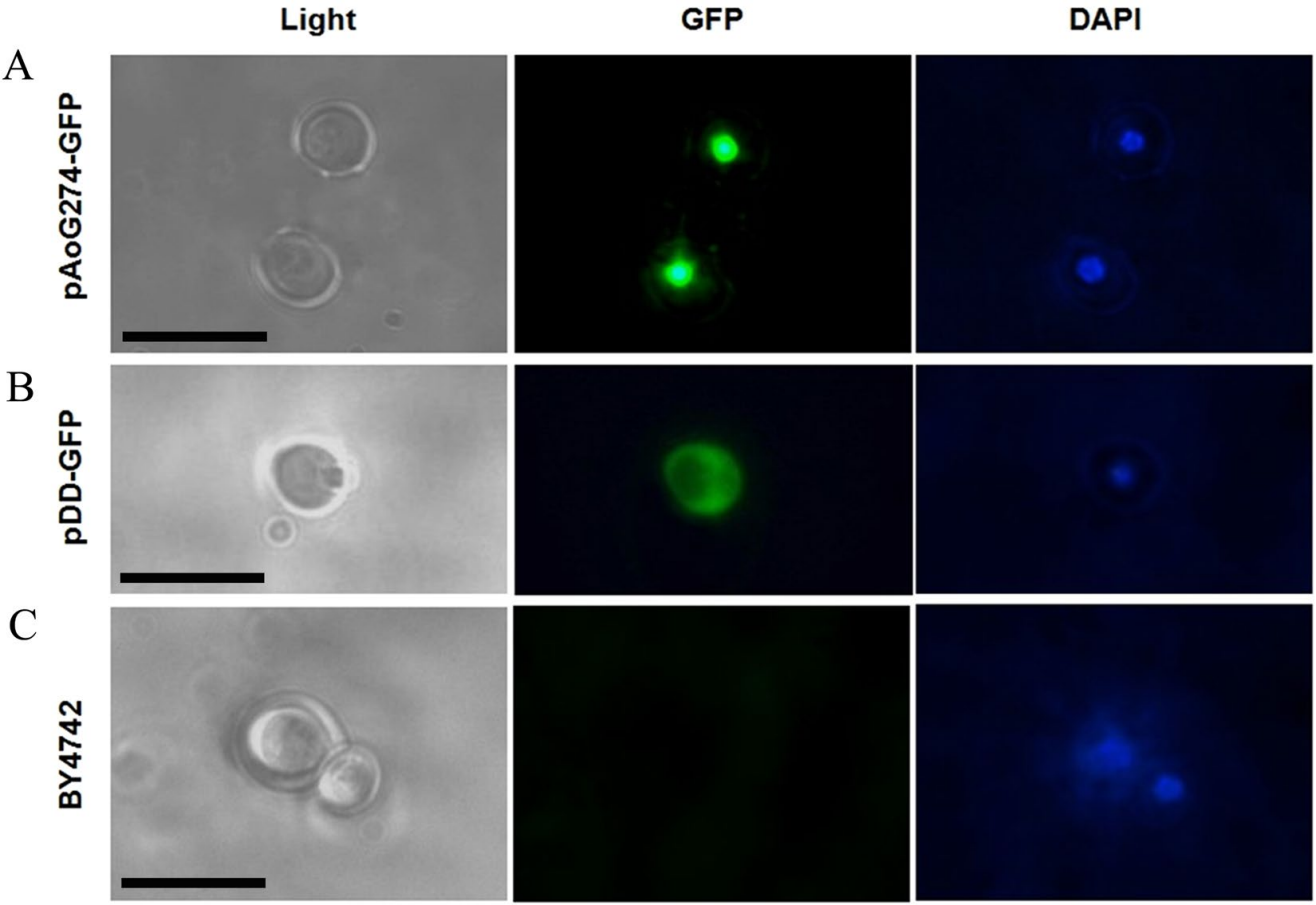
Location of 76g274p-GFP in *Saccharomyces cerevisiae*. (**A**) The genes *76g274* and *gfp* were co-expressed in *S. cerevisiae* BY4742. (**B**) The gene *gfp* was expressed in *S. cerevisiae* BY4742. (**C**) The original strain BY4742. Bar: 10 µm.

In order to investigate the expressional location of the protein 76g274p, the cDNA sequence of *76g274* was cloned into the pDD-GFP as pAo76g274-GFP to express GFP-fused 76g274p. pAo76g274-GFP and pDD-GFP were transformed into *Saccharomyces cerevisiae* (BY4727), respectively. It was observed that only the DAPI-stained nucleus was found in the wild type BY4742 (negative control) (Fig. 5C), and green fluorescent protein (GFP) was uniformly distributed in the cell of strain BY4742::pDD-GFP (Fig. 5B), while GFP signal was centralized in the nucleus of the strain BY4742::pAo76g274-GFP and co-located with DAPI staining (Fig. 5A). Therefore, the gene *76g274* was expressed and located on the nucleus in the model fungus *S. cerevisiae*.

### Expression of gene *76g274* in *A. oligospora* under different conditions

**Figure 6:**
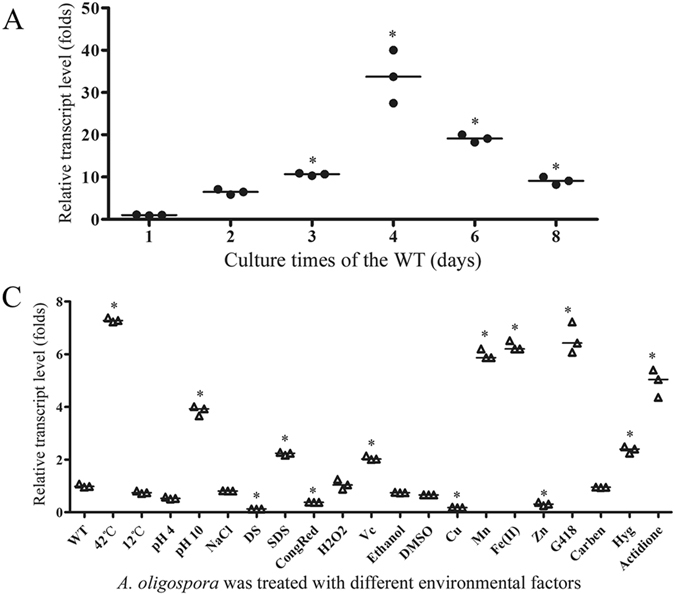
Transcriptional analysis of the gene *76g274* in *A. oligospora* under different conditions. β-tubulin gene (AOL_s00076g640) of *A. oligospora* was used as the internal control. (**A**) The transcription of gene *76g274* in *A. oligospora* during the growth of *A. oligospora* in TG medium. *P < 0.05 versus 1^st^ day. (**B**) The transcription of gene *76g274* under various stress-tolerant conditions (TG broth as basic medium). WT, the control without treated; 42 °C and 12 °C, the mycelia were incubated at 42 °C or 12 °C for 1 h; pH 4 and pH 10, the mycelia were cultured in medium of pH 4 or pH 10 for 4 h; NaCl, the mycelia were treated by 0.5 M NaCl for 4 h; DS, sorbitol (0.5 M); SDS, sodium dodecyl sulfate (0.02%); CongRed, congo red (100 µg/mL); H2O2, H_2_O_2_ (5 mM); Vc, vitamin c (1 mg/mL); Ethanol, 2% ethanol; DMSO, dimethylsulfoxide (2%); Cu, CuSO_4_ (5 mM); Mn, MnSO_4_ (5 mM); Fe(II), FeSO_4_ (5 mM); Zn, ZnSO_4_ (5 mM); G418, geneticin sulfate (300 µg/mL); Carben, carbendazim (300 µg/mL); Hyg, hygromycin B (200 µg/mL); Actidione, 200 µg/mL actidione. The mycelia were treated at above condition for 4 h, respectively. *P < 0.05 versus WT. The horizontal bars depict the median, and data are presented as suggested by Weissgerber *et al*.^58^ and Klaus^59^.

The transcriptional level of gene *76g274* was determined in *A. oligospora* incubated on TG medium at 26 °C. Its transcription was shown to constantly increase to a highest level of 33.7 folds for initial four days, and then decreased with the extension of culture times (Fig. 6A). In addition, the transcriptional level of gene *76g274* was also analyzed under various environmental factors (Fig. 6B). For different temperature treatments, the transcription of gene *76g274* decreased slightly when the mycelium was incubated at 12 °C for 1 h, and sharply enhanced at 42 °C for 1 h compared to the 26 °C environment. Among the pH treatments, its transcription was down-regulated at pH 4.0 but up-regulated by 3.87 folds at pH 10.0 for 4 h. For osmotic stress treatments, the transcription of gene *76g274* was down-regulated by treated with 0.5 M NaCl and sorbitol (DS) for 4 h, respectively, with the most inhibition by DS (a decrease of 88%). For other treatments, the transcription of gene *76g274* was up-regulated by 2.2 folds when treated with 0.02% SDS for 4 h, up-regulated by 2 folds treated by vitamin C (1 mg/mL) for 4 h, and up-regulated by 6.6, 4.9 and 2.5 folds by G418, Hyg, and actidione respectively. In contrast, it was inhibited by Congo red (100 µg/mL), ethanol (2%) and DMSO (2%). The transcription of gene *76g274* was, not influenced by treatment with 5 mM H_2_O_2_ or carbendazim (300 µg/mL) for 4 h. Among the treatments with metals, the transcription of gene *76g274* was significantly inhibited by Cu^2+^ but dramatically increased by Mn^2+^ and Fe^2+^.

### Disruption of *76g274* impairs conidiation in *A. oligospora*

**Figure 7:**
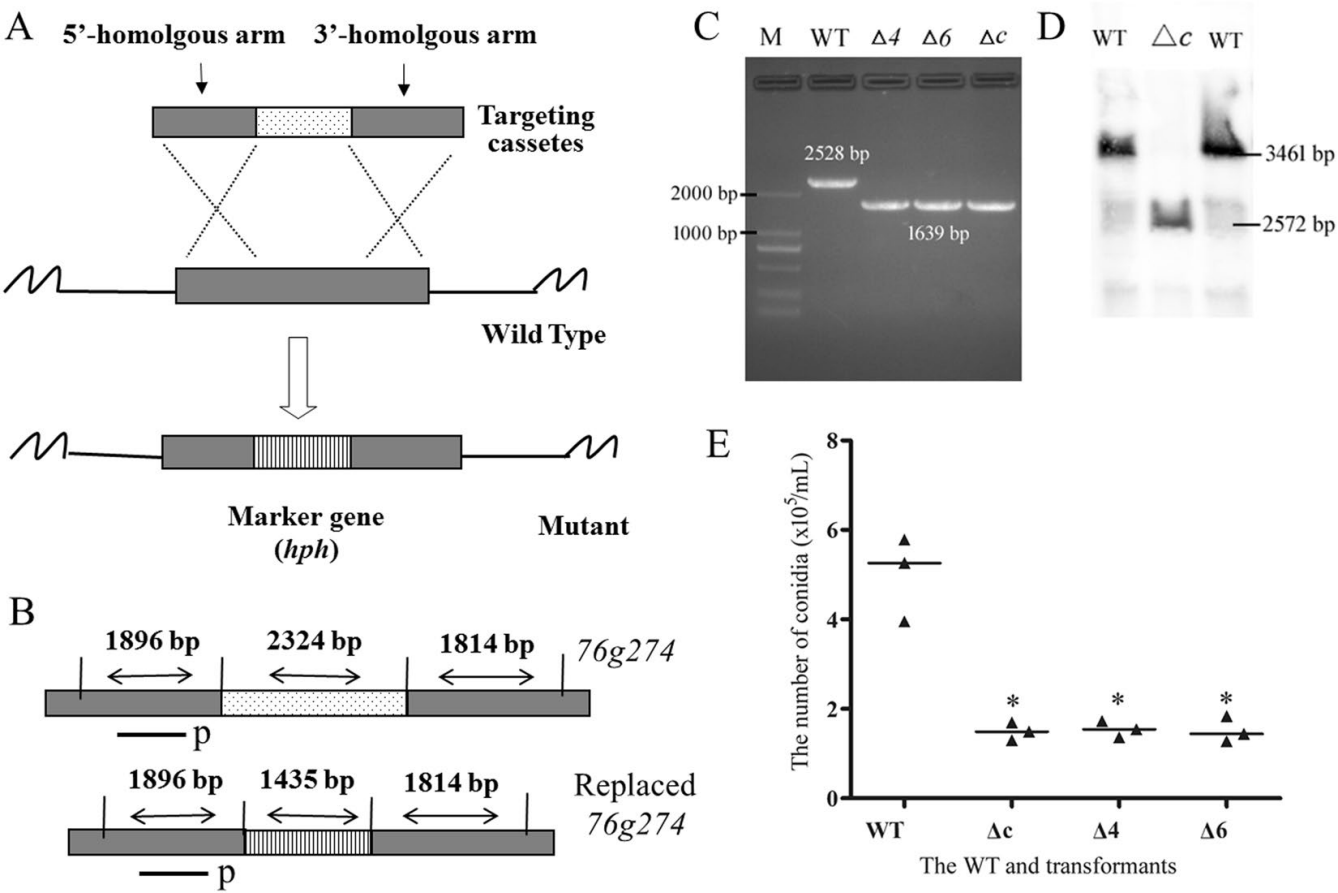
Disruption of gene *76g274* in *A. oligospra* and Southern blotting analysis. (**A**) The sketch map of gene knock-out by homologous recombination. (**B**) Replacement and Southern blotting analysis of gene *76g274*. The length of fragments was marked in figure and p indicated the probe site. (**C**) The PCR verification of WT and three mutants. (**D**) The Southern blotting analysis of WT and mutant Δ*c*. (**E**) Comparison on conidia number of WT and three mutants. *P < 0.05 versus WT. The horizontal bars depict the median, and data are presented as suggested by Weissgerber *et al*.^58^ and Klaus^59^.

The *76g274* gene was disrupted by homologous recombination (Fig. 7A,B) and three transformants (Δ*4*, Δ*6* and Δ*c*) were screened and verified by PCR using primers 274YZ-F and 274YZ-R (Supplementary Table S2) (Fig. 7C), respectively. The growth rate of mutants was faster than the WT strain individually in CMA and WA media, while no obvious difference was observed when growing on PDA medium. Meanwhile, WT and three mutants had no difference in resistance to NaCl (0.3 M), SDS (0.06%) and H_2_O_2_ (15 mM). Moreover, difference on the proteolytic activity was not observed between the WT and mutants, and all three mutants produced traps at the same level as the WT. However, the conidiation of mutants was remarkably inhibited and the sporulation yield of mutants was decreased by about 70% (Fig. 7E).

**Figure 8:**
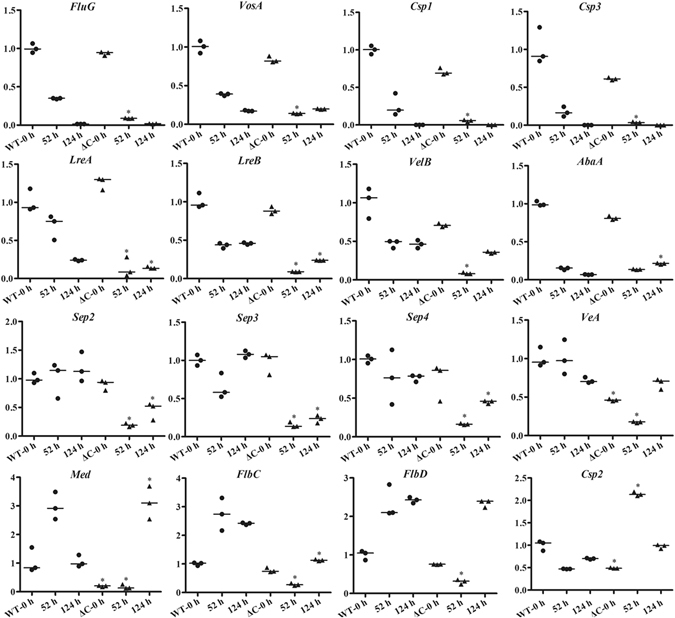
Transcriptional analysis of sporulation-related genes in WT and mutant Δ*c* at different times. The horizontal axis shows the culture times of the WT and mutant Δ*c*, and the vertical axis shows the relative transcriptional levels of each gene at indicated time (0 h, 52 h and 124 h, respectively). The black dots show the WT, and the black triangles show the mutant Δ*c*. The GenBank nos. of sporulation-related genes and their annotations can be found in Supplementary Table S3. β*-tubulin* gene (AOL_s00076g640) of *A. oligospora* was used as the internal control. *P < 0.05 Δ*c* versus WT. The horizontal bars depict the median, and data are presented as suggested by Weissgerber *et al*.^58^ and Klaus^59^.

In order to identify the potential roles of gene *76g274* in *A. oligospora*, the mutant Δ*c* was further confirmed by Southern blotting analysis. The single band hybridizing to the *76g274* gene probe was observed in the WT and transformant Δ*c*, and the sizes of the hybridized bands were consistent with expectation (Fig. 7D). The sporulation-related genes in *A. oligospora* were searched based on the identified homologous genes in the model fungus *Aspergillus nidulans*, such as *AbaA*, *FlbC*, *FlbD*, *FluG*, *LreA*, *LreB*, *MedA*, *VosA*, *VeA* and *VelB* (Supplementary Table S3)^32^. The WT and strain Δ*c* were cultured in PL-4 medium^14^ at 26 °C at 180 rpm for 3 days, respectively. The mycelia of WT and strain Δ*c* were collected by filtration, and then transferred to sterilized plate (6 cm) for conidia production at 26 °C. The results showed that the conidia began to produce after 52 h, and the conidia number stopped to increase at 124 h. The transcriptional level of sporulation-related genes was determined by RT-PCR at 0 h, 52 h and 124 h, respectively (Fig. 8). The transcriptional pattern of partial sporulation-related genes, such as *FluG*, *VosA*, *LreA*, *LreB*, *VelB*, *Csp1* and *Csp3*, was similar between WT and strain Δ*c* during the conidiation. They transcribed at high level at the early stage of sporulation (0 h) and decreased at middle and later stages (52 h and 124 h, respectively). Interestingly, conidiation-specific gene *Csp2* showed differential expressional profile from *Csp1* and *Csp3*. Meanwhile, three septin genes, *Sep1*, *Sep2* and *Sep3* also transcribed similarly at different stages of conidiation, and the transcription of *abaA* on each time point was almost overlapped between WT and strain Δ*c*. Remarkably, transcription of *FlbC*, *FlbD* and *Med* was up-regulated at early and middle stages of sporulation in WT, while they was completely disturbed in strain Δ*c*. In summary, above mentioned results showed *76g274* has positive effects on most tested genes to promote sporulation at different stages.

## Discussion

In this study, the sequenced nematode-trapping fungus *A. oligospora* was used as a model strain, and 37 mutants were obtained using REMI method through protoplast transformation. These mutants showed a high degree of genetic stability. Phenotypic analysis showed that the tested mutants differed from the WT in growth, conidiation, spore germination, trap formation, stress tolerance and nematicidal activity. Most notably, the mutant X5 had an obvious enhancement in conidiation and nematicidal activity, while X13 abolished trap formation and reduced pathogenicity for nematodes. However, the transformation efficiency of the *hph* cassette was lower than in previous reports^25, 26^. In summary, REMI technology was an efficient method for constructing a random mutant library and screening the mutants with phenotypic differences in *A. oligospora*.

The inserted locations of *hph* cassette in transformants X5 and X13 were identified using the genome walking method. The *hph* cassette was inserted into the NCR between genes *78g287* and *78g288* in X13 (Fig. S6), and these two genes were transcribed in opposite directions. The distance between them is 1190 bp, and the location of gene *hph* was 739 bp from *78g287* and 451 bp to *78g288*, respectively, so the *hph* cassette might be inserted into the promoter region of both genes. Similarly, the *hph* cassette was inserted into the NCR of genes *76g273* and *76g274* in X5 (Fig. 2A), and they were also transcribed in opposite directions. The distance between two genes is 8332 bp, and the *hph* cassette is 264 bp from *76g273* and 8068 bp from *76g274*. Moreover, the insertion of pMD-hph was further verified by Southern blotting analysis. We did not observe hybridizing brands with the complete pMD-hph as a probe, likely due to the low efficiency of long probe (>4 kb) in this assay. Fortunately, when we used *hph* cassette as probe, expected single band was observed in the genomic DNA of X5 digested by either HindIII or SalI, and no band was observed in the WT under same condition. The results of southern blotting with two different restriction enzymes suggest a single insertion event of *hph*-contained fragment in X5, although it cannot be excluded that the observed phenotypes in the X5 mutant might not be due to the mapped insertion event. Results from PCR and Southern blotting suggested that X5 was a pure mutant with all nuclei containing the *hph* cassette at the same site. In contrast, some nuclei in X13 had the integrated *hph* cassette while others did not. The *hph* cassette might be located in the promoter region of *76g273*, but the transcriptional level of *76g273* was not influenced. Unexpectedly, the transcription of *76g274* was enhanced and increased by about two-fold (Fig. 3B), so the insertion of *hph* cassette might have split a suppressor of *76g274* and broken its negative regulation for *76g274*.

Interproscan and other analyses of protein 76g274p showed it contains two conserved domains, Zinc finger C_2_H_2_-type/integrase DNA-binding domain and Zn(2)-C6 fungal-type DNA-binding domain. And a zinc-finger protein structure was predicted in conserved domain of 76g274p using Swiss-Model and STRING, which is high similarity to the zinc-finger domain from *Xenopus* TFIIIA^31^. Meanwhile 76g274p shared a low homology to other C_2_H_2_-type protein from other filamentous fungi and had a distant relationship with other fungi (Fig. 4A). Moreover, 76g274p was predicted to locate in the nucleus, which was further verified using co-expression technology in *S. cerevisiae* (Fig. 5). According to above analyses, 76g274p maybe a C_2_H_2_-type transcription factor, and it was located in nucleus in *S. cerevisiae*, while no conserved NLS was predicted in 76g274p using PredictProtein. We speculated that it may contain an unknown NLS, while the sequence of NLS need to be furtherly identified.

These analyses suggested that 76g274p might be a potential C_2_H_2_-type transcription factor. In fact, C_2_H_2_ zinc finger proteins are a major class of transcription factors that play crucial roles in fungal growth, development, various stress responses, and virulence^33–37^. Some C_2_H_2_ zinc finger proteins positively regulated fungal conidiogenesis, conidia germination, appressorium formation^33, 34^, and the production of aerial mycelium^35^. Most of them were also involved in stress responses, such as responses to high concentrations of Ca^2+^ or Na^+^ or high osmolarity. Moreover, many of them were also essential for full virulence^36, 37^. However, some C_2_H_2_ zinc finger proteins were involved neither in fungal development nor in pathogenicity^38^.

The gene *76g274* was very sensitive to the change of environmental factors. Its transcription was significantly stimulated or inhibited when the fungus *A. oligospora* was treated with unfavorable conditions (such as extreme temperatures and pH values, high osmotic pressure, and exposure to heavy metal ions, cell wall-damage agents, peroxide, organics and antibiotics) (Fig. 6B). For example, the transcriptional level of gene *76g274* was enhanced remarkably by high temperature, high pH, several metal ions (Mn^2+^, Fe^2+^) and antibiotics (G418, Hyg and actidione), and its transcription was inhibited significantly by sorbitol, Cu^2+^ and Zn^2+^. The sensitivity of this gene in response to variable environments might be beneficial for the fungus to adapt to changing environments, which also helps explain why some fungi show various colonial morphologies in different media^39^.

In order to identify the potential function of *76g274*, the deletion mutants were constructed using homologous recombination (Fig. 7A). Phenotypic analysis found that the mutant did not differ significantly from the WT in growth, resistance to NaCl, SDS and H_2_O_2_, proteolytic activity, as well as trap formation, but the mutant had sharply reduced sporulation yields (Fig. 7E). Though the strain X5 seemed to have an increase in pathogencity and stress-resistance compared to *76g274*-deficient mutants, it can be explained as the large number of conidia from X5 due to moderate high expression of *76g274* contributed to those phenotypes, because conidia can directly produce traps according previous research and always have stronger anti-stress capacity^10, 40^. Meanwhile, the transcription of the majority of sporulation-related genes was significantly down-regulated during the conidiation in mutant Δ*c* (Fig. 8). In strain X5, the transcription of gene *76g274* was increased by about two-fold, and the sporulation yield was increased by 10.8 folds. These results illustrated that 76g274p might be a novel transcription factor, which positively regulated the conidiation in *A. oligospora*.

There are some shortcomings exist in our present work, such as lacking overexpression and complementation of gene *76g274*. In order to confirm furtherly the function of this gene, much work had been done to construct the *76g274*-overexpressing strain, while we had not obtained stable-overexpressing strain. The genetic manipulation system to overexpress genes is extremely difficult in this fungus, likely partly due to its multinucleic nature. Meanwhile, we also failed to construct complementation of the 76g274 deletion mutant due to available resistant markers for NTF are rather limited. We had attempted common markers used in other fungi, including glufosinate-ammonium, G418, benomyl, aureobasidin A, phleomycin or pyrithiamine, while they are not effective for NTF, so we could not find the second selective marker to perform complementation analysis at present. Moreover, ATMT had also been attempted to construct the mutant library in *A. oligospora* in our laboratory, however no satisfactory result was acquired. In recent years, several methods had been developed and used for the genetic transformation in fungi, for example zinc finger nucleases^41^, TALE-nucleases^42–44^, Δ*ku70*/*ku80*/*Lig4* ^45^. Especially, the CRISPR/Cas9 sysytem^43, 46^ had been used in several fungi for successful genetic manipulation and genome edition, such as *Trichoderma reesei* ^47^. NTF play a vital role in control of plant parasitic nematodes, so it is very necessary to develop an efficient method for genetic manipulation in NTF, which would contribute vastly to understanding the molecular mechanism of NTF infection against nematodes and also to screening high-virulent strains for biological control of nematodes.

## Materials and Methods

### Strains, plasmids and culture conditions

*A. oligospora* strain ATCC24927 used in this study was purchased from American Type Culture Collection (ATCC) and maintained on potato dextrose agar (PDA) at 26 °C^14^. *Saccharomyces cerevisiae* (FY834) was used for recombinational cloning procedure and grown in YPD or YPDA plates. SC-Ura plate was used for selecting colonies of yeast strain FY834 harboring correctly recombined constructs^48^. Plasmids (pRS426 and pCSN44) were maintained in *Escherichia coli* strain DH5а (Takara, Shiga, Japan), respectively. Regeneration medium (PDASS) was used for protoplast regeneration^27^, and PDASS supplemented with 200 µg/mL Hyg (Amresco, Solon, USA) was used for selecting transformants.

*S. cerevisiae* (FY4742) was used for recombinational cloning procedure and grown in YPD; the plasmid pDD-GFP was used to locate the target gene. *Caenorhabditis elegans* (strain N2) was grown in oatmeal medium at 26 °C, and the nematode extract was prepared according to our previous report^14^.

## REMI transformation

The *hph* cassette was amplified from the plasmid pCSN44 with primer set Hph-F and Hph-R (Supplementary Table S2), and the PCR fragment was cloned into vector pMD18-T (Takara, Shiga, Japan), the recombinational plasmid (pMD-hph) (Supplementary Fig. S1) was isolated using Plasmid Mini Kit (Takara, Shiga, Japan). The pMD-hph was digested using restriction enzyme XbaI or SmaI for further use. Protoplasts of *A. oligospora* were prepared and transformed according to methods described previously^24, 27^. Transformants (mutants) were selected initially on PDASS containing 200 µg/mL Hyg and confirmed further by PCR analysis using primers Hph-1F and Hph-1R (Supplementary Table S2), and the genetic stability of transformants was detected according to methods described previously^24, 27^.

### Analysis of resistance of *A. oligospora* and transformants to stress conditions

The TG medium was used as basic medium, NaCl (0.3 M), H_2_O_2_ (30 mM) and SDS (0.05%) were supplemented in TG, respectively. The WT and mutants were incubated respectively on above medium at 26 °C for 7 d, and the growth rate was determined and colonial morphology was observed.

### Vegetative growth and conidia yield comparisons

The mycelial plugs of the WT and mutants of the same size and age were incubated on PDA and TYGA media at 26 °C for 7 days, respectively, the growth rate and morphology of colony were observed and determined. Sporulation capacity of WT and mutants was determined according to our previous report^27^. Each experiment was performed with three biological replicates.

### Protease activity assays

The WT and mutants were incubated on LMZ medium^49^ at 26 °C, respectively. The fermentation liquid was collected after culturing for 6 days. Protease activity was assayed on casein-plate using the method described by previous report^49^.

### Induction of trap formation and virulence assays

Trap formation in *A. oligospora* induced by nematodes and nematode extract according to our previous report^27^. The plate was incubated at 26 °C for 3–4 days, 80–100 adult nematodes were added to the plate. After 12 h, mycelia and traps were observed at specified time intervals under light microscopy (Olympus, Tokyo, Japan). Meanwhile, the number of captured nematodes was counted using a light microscope^27^. The bioassay was performed with three biological replicates.

### Identification of the insertion sites of the *hph* cassette in strains X5 and X13

To identify the insertion site of the *hph* cassette from strains X5 and X13, two nested PCR primers (Supplementary Table 2) were designed according to the nucleotide sequence of the *hph* cassette. The 5′ terminal fragment of the *hph* cassette was amplified using the nested PCR primers and the DNA-Walking Speedup™ Premix Kit (Seegene, Korea), respectively. PCR amplification was performed according to the user’s manual. The PCR product was cloned and sequenced, and the insertion site was identified by blast analysis in the genome of *A. oligospora*. Meanwhile, the mutation site was further identified by PCR amplification using two pairs of primers in X5 and X13, respectively (Supplementary Table 2). Southern analysis was carried out according to the instructions provided by the North2South^®^ chemiluminescent hybridization and detection kit (Pierce, Rockford, USA). The probe was amplified using pair X5-S1/X5-S2 (Supplementary Table S2), and restriction enzymes SalI and HindIII were used to digest the genomic DNA for Southern analysis.

### Sequence and phylogenetic analysis of 76g274p

The sequence of 76g274p (EGX50199) in *A. oligospora* was downloaded from the GenBank. The theoretical isoelectric point (pI) and molecular weight of 76g274p were calculated using the pI/MW tool^50^, and conserved functional domains were analyzed via the InterPro Web site^28^. Moreover, the structure of 76g274p was predicted by PredictProtein^51^, and its tertiary structure was analyzed by Swiss-Model^52^ and Phyre2^53^. Meanwhile, the protein-protein interaction was predicted using STRING 9.05^54^.

The amino acid sequence of 76g274p was retrieved and its analogues in different fungi were downloaded from GenBank. A neighbor-joining tree was constructed using the Mega 5.1 software package^55^.

### Quantitative PCR (RT-PCR) analysis

Primers were designed and synthesized based on the DNA sequences of target genes, including *76g273* and *AOL_s00076g274* (Supplementary Table S2) and primers of sporulation-related genes in *A. oligospora* were also designed using Primer3 (Supplementary Table S3). Total RNA was extracted with Trizol Reagent (Invitrogen, Carlsbad, California), purified with a RNeasy Mini Kit (Qiagen, Valencia, Califonia), and then reverse transcribed with PrimeScript RT reagent Kit (Takara, Shiga, Japan) by following the manufacturer’s instructions. RT-PCR was conducted according to our recent report^56^. β-tubulin gene (AOL_s00076g640) of *A. oligospora* was used as the internal control. RT-PCR was performed with three biological replicates for each gene.

### Locolization analysis of the gene* 76g274* in *S. cerevisiae*

The cDNA sequence of *76g274* without terminator (TAA) was amplified using primer pair 76g274F/76g274R, and it was inserted into the linearized vector pDD-GFP (digested by *SmaI*), the final vector pAoG274-GFP was transformed into *S. cerevisiae* (BY4742), and tranformants were screened on SC-Ura plate. The strain BY4742, BY4742::pDD-GFP and BY4742::pAoG274-GFP were cultured in YPD at 30 °C for 12 h, three strains were collected, respectively, and observed under fluorescence microscope.

### Deletion of the gene *76g274*

The gene *76g274* replacement construct was generated using a modified yeast cloning procedure^48, 57^. Two fragments corresponding to the 5′ and 3′ of the gene *76g274* were amplified with primer sets 274–5 F/274-5 R and 274-3 F/274-3 R (Supplementary Table S2), respectively. The two fragments were co-transformed into yeast strain FY834 along with the *hph* cassette and gapped yeast shuttle vector (pRS426). The final disruption vector (pRS426-76g274-hph) was recovered by transformation into *E. coli* DH5a. Protoplast of *A. oligospora* was prepared and transformed according to our recent report^27^. Transformants were selected on PDASS containing 200 µg/mL Hyg, and further confirmed by PCR and Southern blot analyses. Southern analysis was carried out according to above description, and the probe was amplified using primer pair 274-S1/274-S2 (Supplementary Table S2), and restriction enzyme BamHI was used to digest the genomic DNA.

### Statistical analysis

Medians were calculated as recommended for biomedical research^58, 59^. One-way analysis of variance (ANOVA) followed by Tukey’s multiple comparison test is indicated when used, (*P* < 0.05). All statistical analyzes were made using GraphPad Prism version 5.00 for Windows (GraphPad Software, San Diego, California, USA).

## Electronic supplementary material

Supplementary Information
