# Supplementary material for: Random mutagenesis analysis and identification of a novel C2H2-type transcription factor from the nematode-trapping fungus Arthrobotrys oligospora
Source: Sci Rep. 2017 Jul 17;7:5640. doi: 10.1038/s41598-017-06075-5 (PMC5514059; doi:10.1038/s41598-017-06075-5)

Random mutagenesis analysis and identification of a novel C_2H_2-type transcription factor from the nematode-trapping fungus *Arthrobotrys oligospora*

Dewei Jiang[1,2,#], Jing Zhou[1,3,#], Guizhen Bai[1], Xinjing Xing[1], Liyan Tang[1], Xuewei Yang[1], Juan Li[1], Ke-Qin Zhang[1,*], Jinkui Yang[1,*]

[1]State Key Laboratory for Conservation and Utilization of Bio-Resources in Yunnan, Yunnan University, Kunming 650091, P. R. China.

[2]Key Laboratory of Animal Models and Human Disease Mechanisms of the Chinese Academy of Sciences & Yunnan Province, Kunming Institute of Zoology, Chinese Academy of Sciences, Kunming 650223, P. R. China.

[3]Panzhihua Institute for Food and Drug Control, Panzhihua 617000, P. R. China.

[#]Dewei Jiang and Jing Zhou contributed equally to this work.

*To whom correspondence should be addressed. Tel: 86-871-65032538; Fax: 86-871-65034838. E-mail: kqzhang1@ynu.edu.cn (Ke-Qin Zhang); jinkui960@ynu.edu.cn (Jinkui Yang).

Supplementary tables

Table S1. The endonuclease-induced damage rate of protoplast and the mutants generated by REMI transformation.

	R^0	R^{10}	R^{30}	R^{60}	XbaI	SmaI
Protoplasts	100 μL	100 μL	100 μL	100 μL	100 μL	100 μL
Linearized vector	—	—	—	—	2 μg	2 μg
Enzyme concentration	0 U	10 U	30 U	60 U	30 U	30 U
Colonies	600+	500+	400+	108	41	7
Damage rate of protoplast	0	~15%	~30%	>80%	—	—

Table S2. List of PCR primers used in this study

Primers	Sequences (5'-3')	Description
Hph-F	GTCGGAGACAGAAGATGATATTGAAGGAGC	Amplify the *hph* cassette
Hph-R	GTTGGAGATTTCAGTAACGTTAAGTGGAT	
Hph-1F	ACCTGCCTGAAACCGAACTG	Verify the transformant containing *hph* cassette
Hph-1R	TTCTGCGGGCGATTTGTGT	
Va1	CGGCTCCAACAATGTCCTGA	Amplify the 5' fragment of the *hph* cassette in X5 and X13
Va2	TGGTTGGCTTGTATGGAGCAGCAGAC	
X5inserF	TTGCCGTCAGTATCCAGTCC	Identify the mutation site in X5
X5inserR	CCACTTACCAGCCTTCGTGA	
X13inserF	TTCACATAGAAAGCAGCCGC	Identify the mutation site in X13
X13inserR	GCAGAACGAAGTGTAGATGC	
Tub1	CCACCTTCGTCGGTAACTC	RT primer for the β-tubulin gene
Tub2	TCGTCCATACCCTCACCAG	
273F	GTTGTATCTGGGCCTGTGCTA	RT primer for the gene *76g273*
273R	TGTCGATGTATTCGAGTGCCA	
274F	CAAATGCGACCCACCTTTCG	RT primer for the gene *76g274*
274R	AGGAAAATCTGGGAAGGCCG	
287F	TGGAACATGGACTATGGCCG	RT primer for the gene *78g287*
287R	GCAACCCAGATTACGGGACA	
288F	CCACTGTAGCAAGTGATCCGA	RT primer for the gene *78g288*
288R	CCGACGAGGGTAGATGTCAGT	
76g274F	TCGACGGATTCTAGAACTAGTGGATCCCCCAT GCACCAGCCTCCCGTCAG	Amplify the gene *76g274* for constructing the GFP-fusion plasmid
76g274R	AAATTGACCTTGAAAATATAAATTTTCCCCCT CCTTTTTCACAATTTCCG	
274-5F	GTAACGCCAGGGTTTTCCCAGTCACGACGTATCG GACTGTGACTTGCG	Amplify the 5′ flank of *76g274*
274-5R	ATCCACTTAACGTTACTGAAATCTCCAACCGAGT AAAGGCAGAACGGTC	
274-3F	CTCCTTCAATATCATCTTCTGTCTCCGACTCTCTT GACTTACCCGCTTGT	Amplify the 3′ flank of *76g274*

274-3R	GCGGATAACAATTTCACACAGGAAACAGCTTCT TTCCCACGGAGCACTG	
Cross-F	TTGCTGTGCTTTCCTTGG	Amplify the full sequence of gene *76g274*
Cross-R	ATAGACGACAACCTAAGCG	
274YZ-F	CCACGTCTTATTTCGGTGCC	Verify the Δ*76g274* strains
274YZ-R	GTCGAGGGGTAGAGGAGGTA	
274-S1	CACCAGTACCCACCCTCTTT	Southern blotting probe of gene *76g274*
274-S2	AGCTAGCTGATGTATGGCGT	
X5-S1	GACAGAAGATGATATTGAAGGAGC	Southern blotting probe of gene *hph* cassette
X5-S2	GATTTCAGTAACGTTAAGTGGAT	

Table S3. List of primers for RT-PCR of sporulation-related genes in *A. oligospora*.

Genes	Annotated	Primers	Sequences (5'-3')
tubA (AOL_s00076g640)	β-tubulin	tubA-F	CCACCTTCGTCGGTAACTC
		tubA-R	TCGTCCATACCCTCACCAG
AOL_s00210g120	Transcriptional regulator Medusa (Med)	210g120-qF	TTCTTGAAGCTCTCGTCGGT
		210g120-qR	CTTAGCCTTGGAGACGGTCA
AOL_s00043g361	Protein fluG (FluG)	043g361-qF	CCCGCCTCATGAAGAAATCG
		043g361-qR	AATCTCTTGTTCCGCGATGC
AOL_s00054g700	VosA	054g700-qF	CCAGTGCACAAATGAGGAGG
		054g700-qR	TATGCGCATCGTGATAGGGT
AOL_s00054g811	VelB	054g811-qF	TCGACGGCCTATTACTCCAC
		054g811-qR	TGAGGGAGAAGTTGCGGAAT
AOL_s00080g63	AbaA	080g63-qF	TTTATGCGCCTTGTCGTAGC
		080g63-qR	TTGGCTAGGTGGTCTGTACG
AOL_s00007g157	FlbC	007g157-qF	CTCTCCGGCAAAGACAATCG
		007g157-qR	TAGCTGTTTGTGGATGCTGC
AOL_s00097g46	FlbD	097g46-qF	GTATTCCCATCAAACCCCGC
		097g46-qR	GCAAGAGGGCTGTGTAGGTA
AOL_s00083g487	LreA	083g487-qF	AGGAGGTGTAATGCAAGGCT
		083g487-qR	TGTCGGATCCAAGCTGTCAT
AOL_s00169g18	VeA	169g18-qF	ATGGTGGGCATTATGTTCGC
		169g18-qR	AAGAACGATGTGGGACCTCC

AOL_s00080g93	LreB	080g93-qF	CCCTCTGCATTTCGACATGG
		080g93-qR	CTGCCACTCCTACTAGTCCG
AOL_s00007g374	Conidiation-specific	007g374-qF	ATGCTCGCCTAAAACCCTCT
	protein (Csp1)	007g374-qR	CAACCCGGGAATGCTTCTTC
AOL_s00079g472	Conidiation-specific	079g472-qF	GCCCTTTGGAATGACACCTG
	protein (Csp2)	079g472-qR	TACGTCAAGTGGCTGGTCTT
AOL_s00081g178	Conidiation-specific	081g178-qF	CCTCAAAGCCAGCATTCGTT
	protein (Csp3)	081g178-qR	TGTTGAAGCAGCCGGTAGTA
AOL_s00215g893	Cdc1, septin 2	215g893-qF	ATACCGCCAACACCCTCTAC
	(Sep2)	215g893-qR	AACCATCTTCATCTCGGCCT
AOL_s00188g32	Cdc1, septin 3	188g32-qF	ATGAGCCGGTTCGATCTACC
	(Sep3)	188g32-qR	ACTTGGTCTCCGTATCCTGG
AOL_s00112g7	Cdc1, septin 3	112g7-qF	TAACCGTGTGCATGCTCTTC
	(Sep4)	112g7-qR	GACTGGGATGACATTGACGC

Supplementary figures

Fig. S1 The schematic map for constructing the plasmid pMD-hph. A. The map of plasmid pCSN44, which contains the *hph* cassette. B. The *hph* cassette consists of the encoding region, promoter and terminator. C. The electrophoretic map of the plasmid pMD-hph. M, DNA marker, ladders are 5 kb, 3 kb, 2 kb, 1.5 kb, 1 kb, 750 bp, 500 bp, 250 bp and 100 bp; V, the circular plasmid pMD-hph; Lane 1-6, the plasmid pMD-hph was digested using single restriction enzyme, including XbaI, KpnI, SmaI, SacI, SalI and HindIII. D. The map of cloning vectors pMD18-T and pMD19-T.

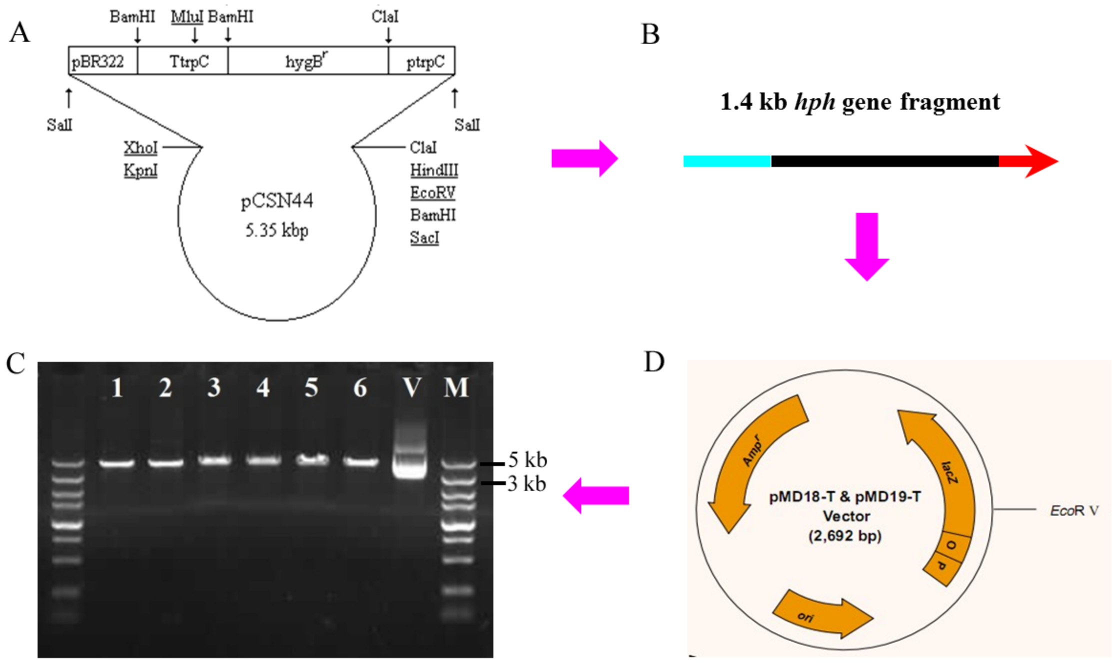

Fig. S2 Comparison of conidial germination of WT and mutants. A. The conidial germination of WT, and only a germination tube was formed from a single conidium. B. Two germ tubes were produced from a single conidium. Scale bar: 10 μm.

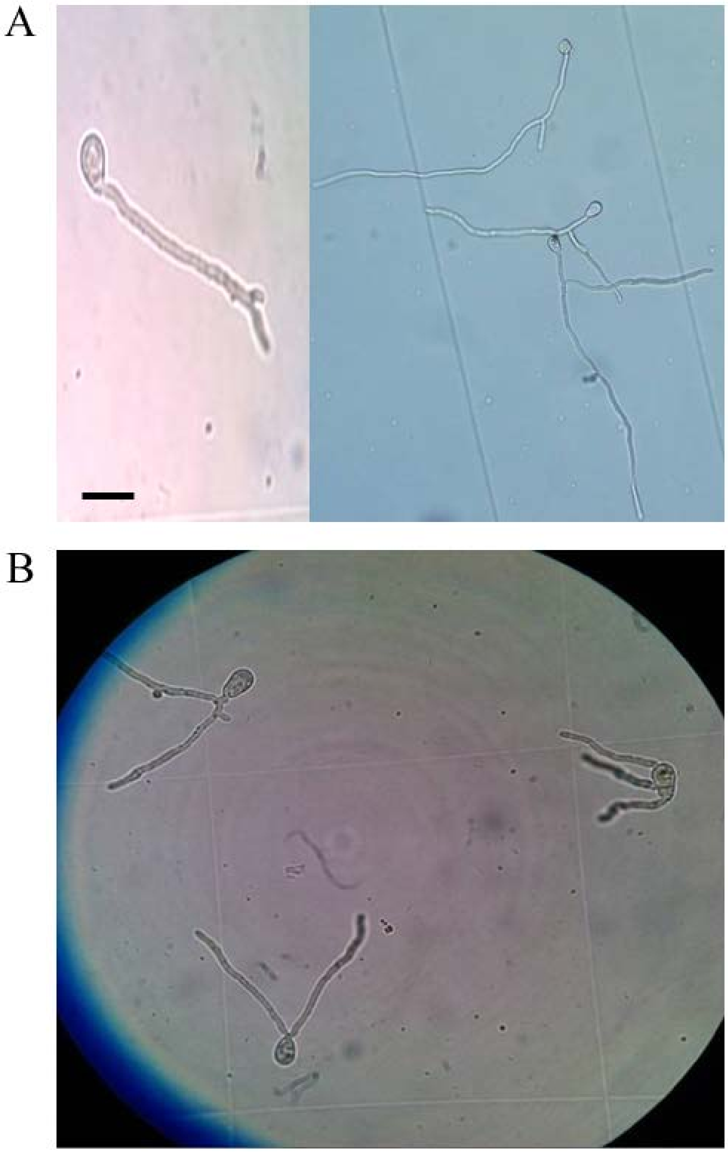

Fig. S3 The proteolytic activities of WT and nine transformants were determined on casein-plates. The red holes showed the negative control (un-inoculated LMZ medium).

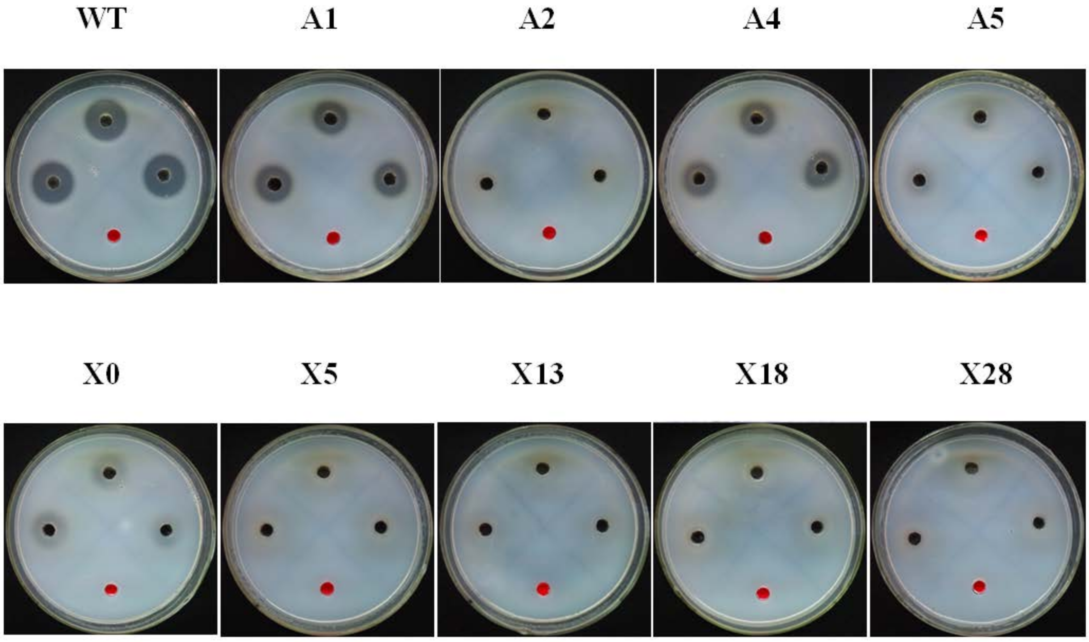

Fig. S4 Trap formation of WT and X13 on WA plate after induced by nematodes for 24 h.

A, the WT; B, the strain X13. Scale bar: 50 μm.

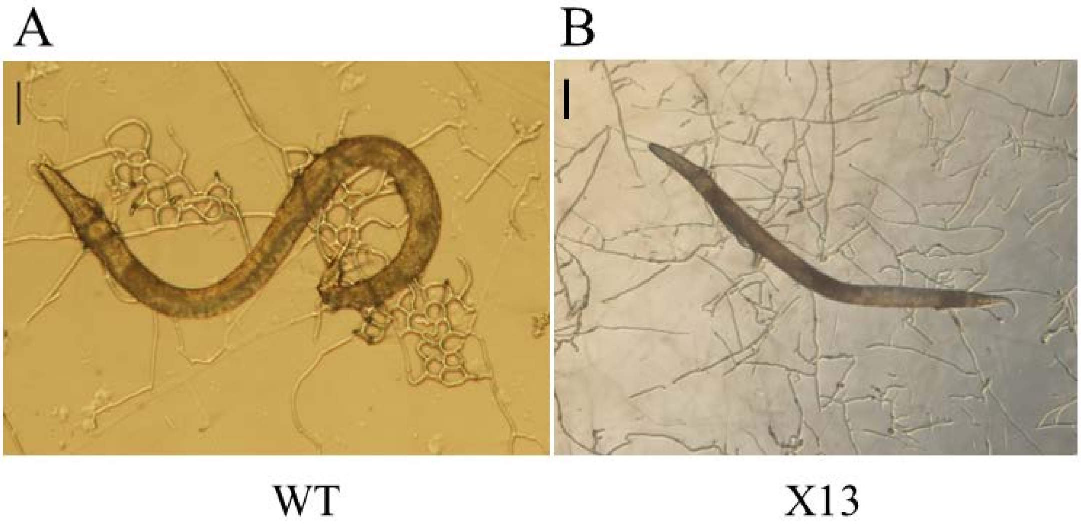

Fig. S5 Growth of WT and transformants on TG plates supplemented with different reagents (NaCl, SDS and H_2O_2) for 7 days at 26 ºC. The concentration of NaCl, SDS and H_2O_2 is 0.05%, 30 mM, and 0.3 M, respectively.

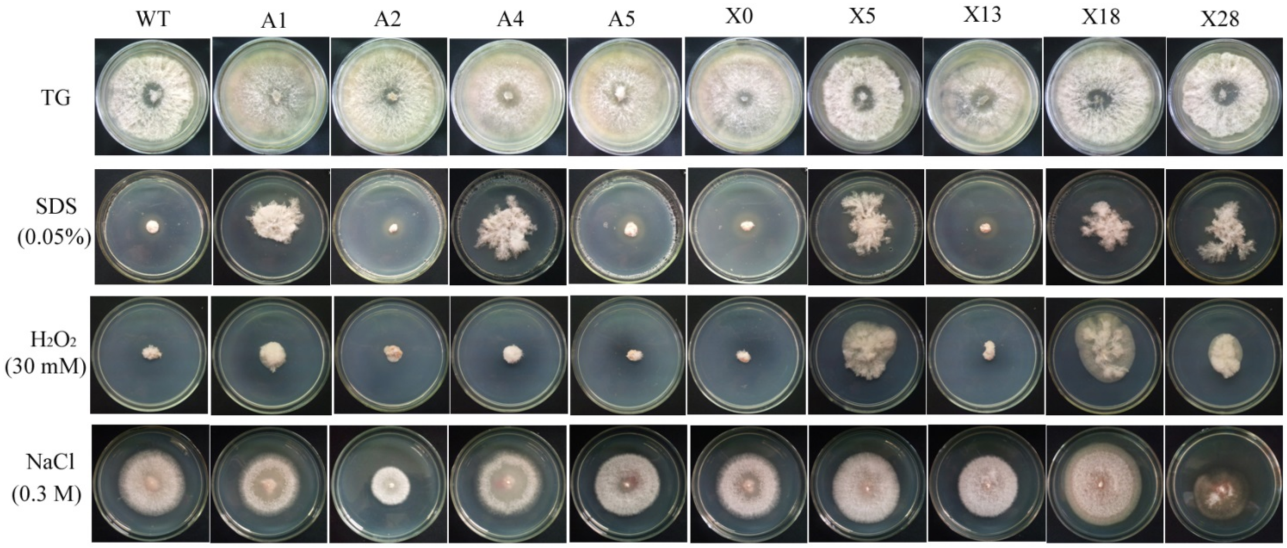

Fig. S6 Identification and verification of the insertion of the *hph* cassette in transformants X13. A. The sketch map of the insertion of the pMD-hph in transformants X13. B. Verification of the insertion of the *hph* cassette in transformants X13 by PCR amplification, the primers X13inserF and X13inserR used for PCR amplification could be found in Supplementary Table S2. M^{5k}, DNA marker, ladders are 5 kb, 3 kb, 2 kb, 1.5 kb, 1 kb, 750 bp, 500 bp, 250 bp and 100 bp.

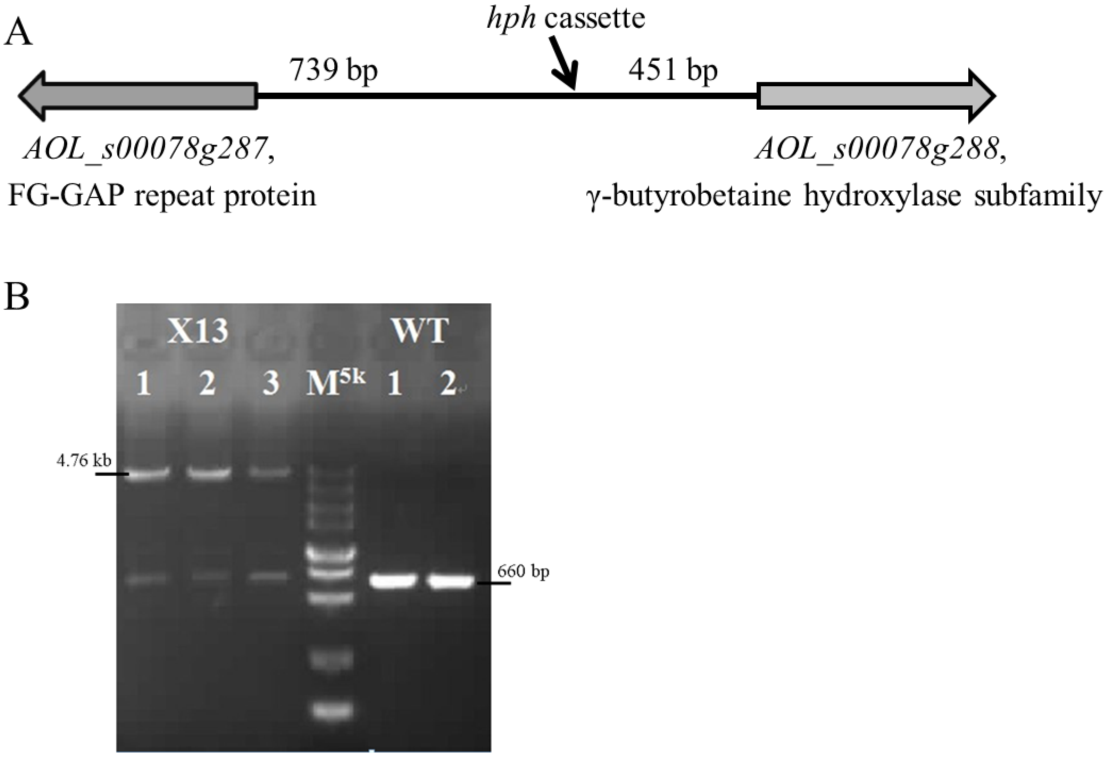

Supplement: Supplementary file 1 — Supplementary Information [file 41598_2017_6075_MOESM1_ESM.pdf]
